# A selective small-molecule inhibitor of c-Met suppresses keloid fibroblast growth in vitro and in a mouse model

**DOI:** 10.1038/s41598-021-84982-4

**Published:** 2021-03-09

**Authors:** Min-Ha Choi, Jinhyun Kim, Jeong Hyun Ha, Ji-Ung Park

**Affiliations:** Department of Plastic and Reconstructive Surgery, Seoul National University Boramae Hospital, Seoul National University College of Medicine, 5 Gil 20, Boramae-Road, Dongjak-Gu, Seoul, 07061 Republic of Korea

**Keywords:** Skin diseases, Health care

## Abstract

Keloids, tumor-like lesions that result from excessive scar formation, have no definitive treatment modality. Activation of c-mesenchymal-epithelial transition factor (c-Met) promotes cell proliferation and survival. Selective c-Met inhibitors, such as PHA-665752, may attenuate the activity of keloid fibroblasts and reduce keloid formation. Here, we aimed to evaluate the effect of PHA-665752, a second-generation selective small-molecule inhibitor of c-Met, on human keloid fibroblasts in vitro and in a mouse model. We performed in vitro cytotoxicity assays, scratch tests, western blotting, and immunofluorescence on human keloid fibroblasts. We also injected human fibroblasts into severe combined immunodeficient mice and measured the degree of nodule formation and skin histologic characteristics. We found that keloid fibroblast migration was inhibited by PHA-665752. Inhibitor treatment was also associated with lower expression of members of the hepatocyte growth factor/c-Met pathway, and lower fibroblast activity and collagen synthesis. In the in vivo experiments, PHA-665752—treated mice had lower nodule volumes and weights, accompanied by less inflammatory cell infiltration and collagen deposition, than those in control mice. These findings showed that although an in vivo model may not accurately represent the pathophysiology of human keloid development, PHA-665752 suppressed keloid fibroblast activity by inhibiting the c-Met—related tyrosine kinase pathway.

## Introduction

Keloid scars are benign but locally aggressive fibroproliferative growths formed during cutaneous healing after injury^1,2^. The lesions can form on any area of skin, and may cause pain and functional impairment. In most cases, the affected sites are aesthetically disfiguring; keloids can cause emotional distress and mental health problems, such as self-image deterioration.

Historically, surgical excision has been the main method to remove keloids. However, keloid recurrence and unsuccessful therapy have prompted the use of other treatment modalities. Intralesional corticosteroid injection improves scar appearance by reducing collagen synthesis, but can produce unsatisfactory results due to skin atrophy and telangiectasia^3^. Adjuvant radiation therapy is associated with organ fibrosis and the potential for malignant transformation. Moreover, medications such as interferons, tacrolimus, and 5-fluorouracil have been considered for keloid treatment, but they remain experimental in this context. Thus, there is no single successful therapeutic regimen for the treatment of keloid scars with a low recurrence rate. The development of a better treatment protocol would dramatically benefit individuals with keloids^4,5^.

The etiology of keloid formation is incompletely understood. Although the mechanism is unclear, genetic predisposition plays a role^6^. Keloid formation is associated with altered growth factor regulation and subsequent abnormal collagen production. According to previous studies, transforming growth factor beta (TGF-β), insulin-like growth factor I, and interleukins are overexpressed in keloid tissues^4–9^. Vascular endothelial growth factor (VEGF) expression is also elevated in keloid tissues and appears to promote keloid growth^3,8^.

c-mesenchymal-epithelial transition factor (c-Met) is a transmembrane tyrosine kinase that acts as a receptor for hepatocyte growth factor (HGF). The HGF/c-Met pathway activates the downstream extracellular signal-regulated kinase (ERK) and phosphatidylinositol 3-kinase (PI3K) pathway, eventually driving cell proliferation and motility^8,10^. c-Met and HGF are dysregulated in cancer cells, and they are believed to contribute to tumor invasion, making the pathway an attractive candidate for targeted cancer therapy^7,9,11^. Phosphorylated c-Met also plays a role in keloid pathogenesis^12–14^. Selective small-molecule inhibitors of c-Met inhibit angiogenesis and tumorigenicity, and have cytoreductive effects, in cancer models. Therefore, agents blocking the c-Met pathway are attractive candidates for the treatment of keloid scars. SU-11274, a first-generation c-Met inhibitor, suppressed keloid fibroblast proliferation, motility, and viability, but was unsuitable for clinical application^7,13^. PHA-665752, a second-generation selective small-molecule inhibitor of c-Met, has more than 50-fold greater selectivity for c-Met than that of other tyrosine and serine/threonine kinases^14^. It has better potency than that of SU-11274 and its effectiveness has been investigated in cancer studies, making it a better candidate for in vivo studies^15,16^.

In this study, we describe the effects of the selective small-molecule c-Met inhibitor PHA-665752 on keloid tissues in vitro and in vivo.

## Results

### Expression of c-Met in keloids

**Figure 1:**
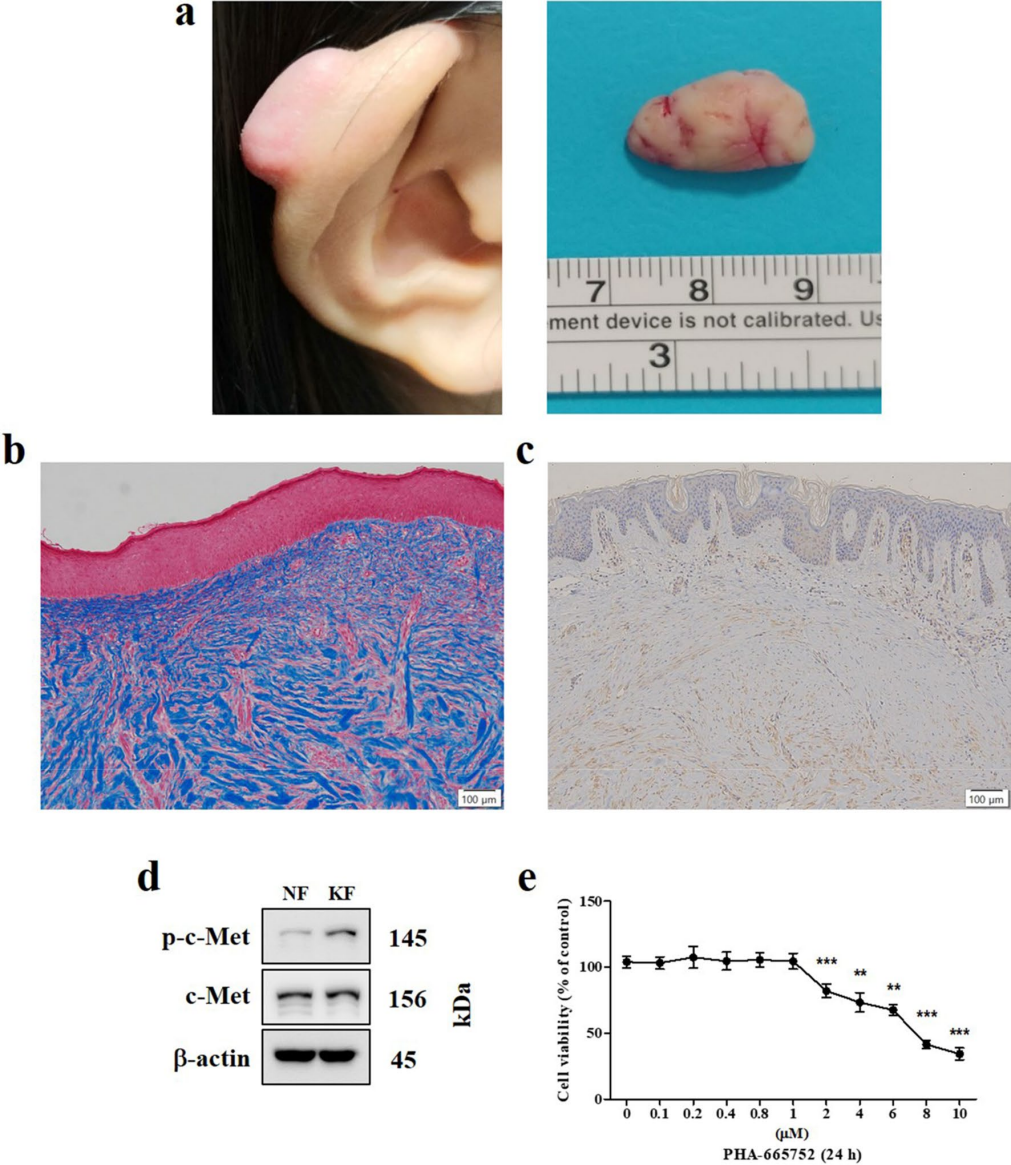
Clinical and histological features of human keloids. (**a**) A human keloid from a human ear. (**b**) Intralesional excisions were histopathologically characterized by MT staining (Original magnification: 100 × , scale bar = 100 µm). (**c**) Immunohistochemical staining of c-Met in human keloid tissue (Original magnification: 100 × , scale bar = 100 µm). (**d**) Protein expression of c-Met and p-c-Met in human keloid-derived fibroblast and normal fibroblast. Full-length blot images are presented in Supplementary Fig. 2a. (**e**) Cell viability and cytotoxicity of PHA-665752 in the CCK-8 analysis (**represents *p* < 0.005, ***represents *p* < 0.0001). *KF* Keloid fibroblast, *NF* Normal fibroblast, *OD* Optical density.

We confirmed the activation of c-Met in human keloid tissue and human keloid-derived fibroblasts using intralesional keloid excision samples harvested from patients (Fig. 1a). Masson’s trichrome (MT) staining revealed that collagen fibrils were randomly distributed in the samples, and mainly located in the dermal aspect (Fig. 1b). We also found that c-Met was expressed in keloid tissue (Fig. 1c), and phosphorylated c-Met (p-c-Met) expression was higher in keloid fibroblasts than that in normal fibroblasts (Fig. 1d). The viability of keloid fibroblasts was significantly lower after treatment with 2, 4, 6, 8, and 10 μM PHA-665752 than that of control-treated keloid fibroblasts. The half maximal inhibitory concentration of PHA665752 for keloid fibroblasts was approximately 7.9 μM (Fig. 1e).

### The effects of PHA-665752 on HGF/c-Met signaling in keloids

**Figure 2:**
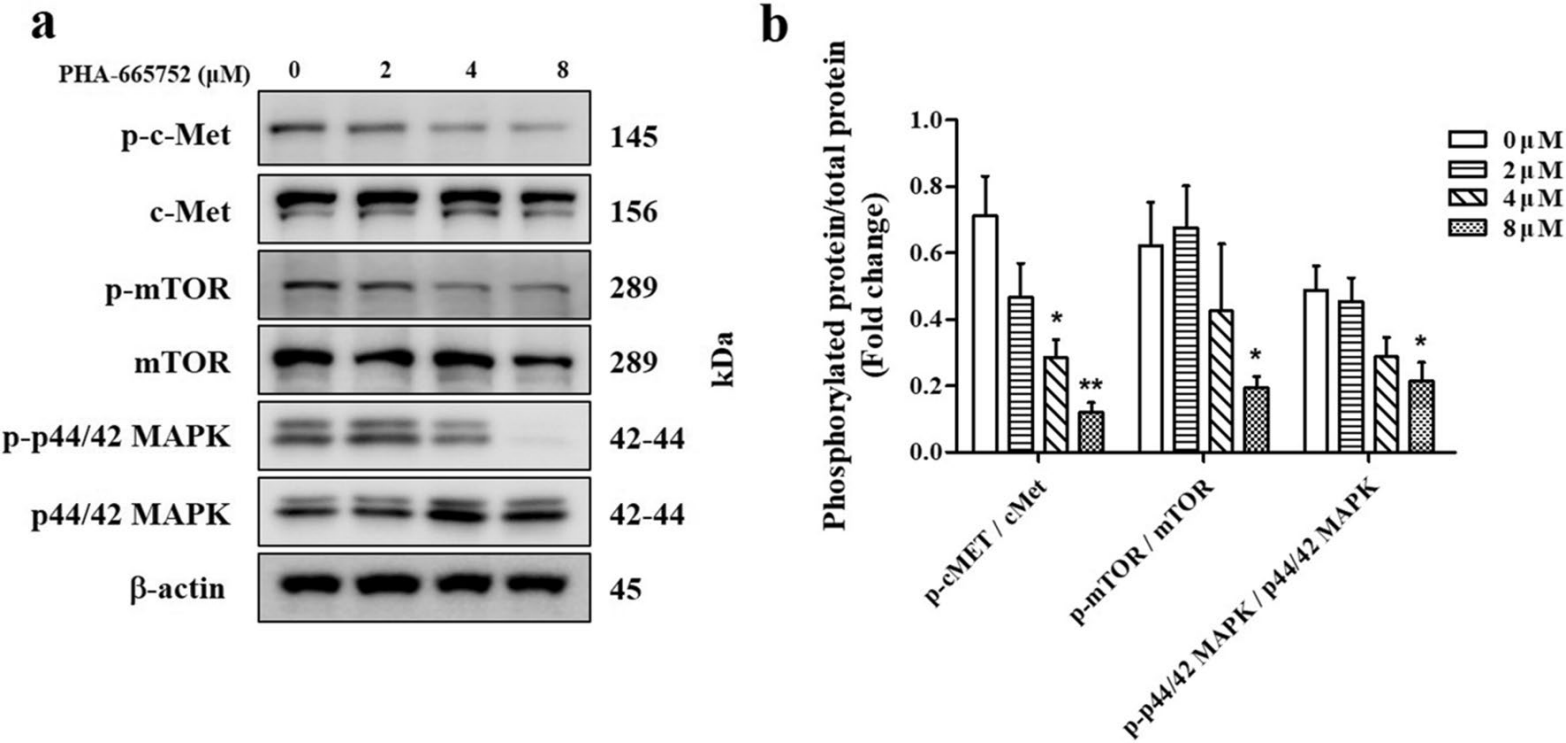
Inhibition of the HGF/c-Met signaling pathway in keloid fibroblasts treated with PHA-665752. (**a**) Western blot of expression of p-c-Met, p-mTOR, and p-p44/42 MAPK in keloid fibroblasts treated with the indicated concentration of PHA-665752. Full-length blot images are presented in Supplementary Fig. 3a. (**b**) Relative protein levels (*represents* p* < 0.05, **represents *p* < 0.005).

Cells were treated with increasing doses (0, 2, 4, and 8 μM) of PHA-665752 for 24 h (Fig. 2a). The level of p-c-Met was significantly lower in cells treated with 4 μM and 8 μM PHA-665752 (0.28 ± 0.14 and 0.12 ± 0.08, respectively) than that in controls (0.71 ± 0.30). The percentage of cells expressing phosphorylated mammalian target of rapamycin (mTOR) was more than 3.2 times lower in cells treated with 8 μM PHA-665752 (0.19 ± 0.08) than that in controls (0.62 ± 0.32). Similarly, the expression of phosphorylated p44/42 MAPK was lower in cells treated with 8 μM PHA-665752 (0.22 ± 0.14) than that in controls (0.49 ± 0.18) (Fig. 2b).

### Keloid fibroblast migration assay after treatment with PHA-665752

**Figure 3:**
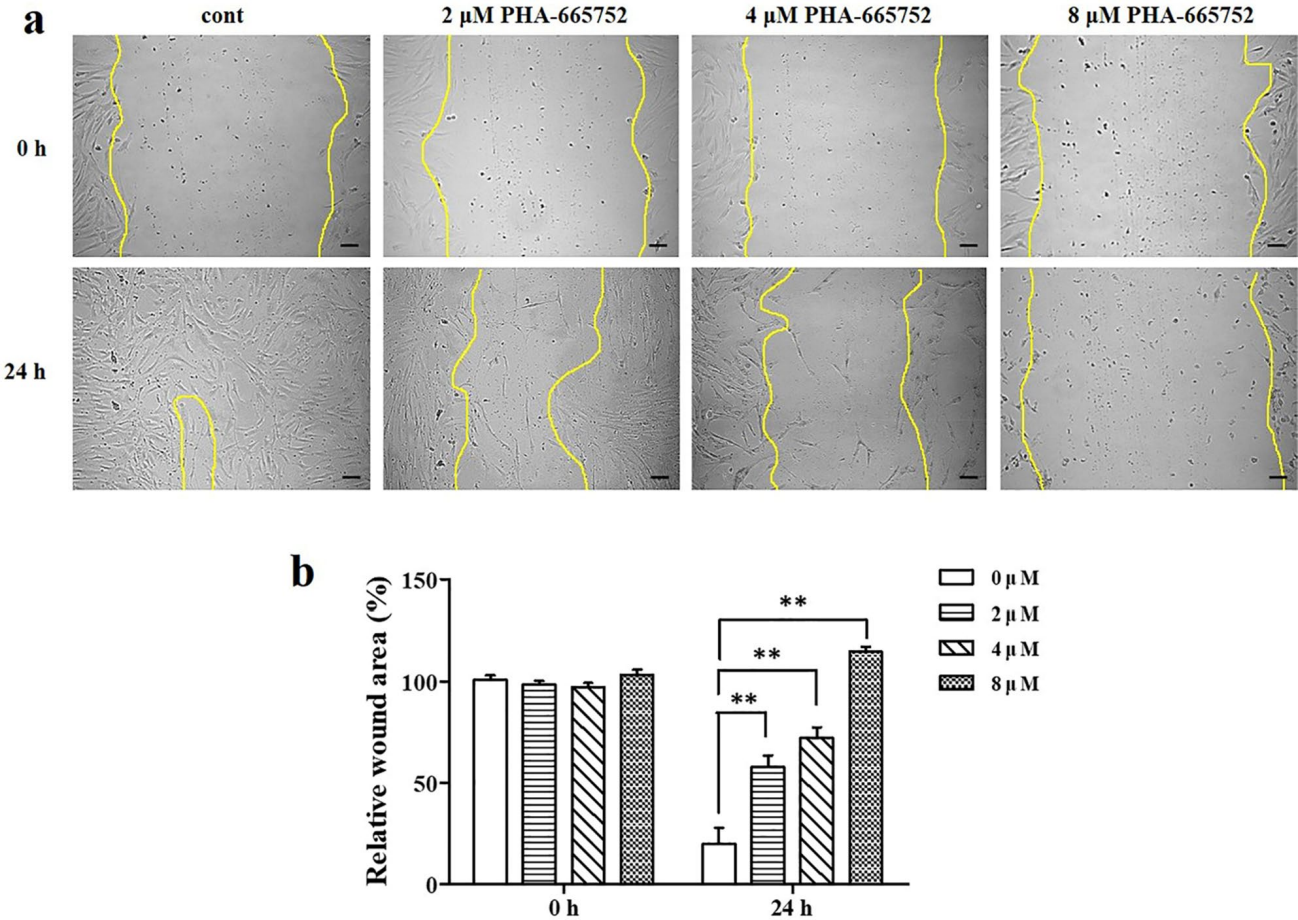
PHA-665752 inhibits the migration of human keloid-derived fibroblasts. (**a**) Keloid fibroblasts were grown to confluence on 24-well plates and treated with 3 different concentrations of PHA-665752 for 24 h (Original magnification: 100 × , Scale bar = 100 µm). (**b**) Quantitative analysis of wound closure (**represents *p* < 0.005).

The cells treated with 2, 4, and 8 μM PHA-665752 showed less migratory ability after 24 h than that in the untreated cells (Fig. 3a). In the scratch assay, we observed significantly larger gaps between cells in cultures treated with 2, 4, and 8 μM PHA-665752 (57.95% ± 9.58%, 72.21% ± 5.86%, and 114.69% ± 6.27%, respectively) than that between cells in the control culture (23.91% ± 18.5%) (Fig. 3b).

### Effect of PHA-665752 on collagen accumulation-related extracellular matrix components in keloids

**Figure 4:**
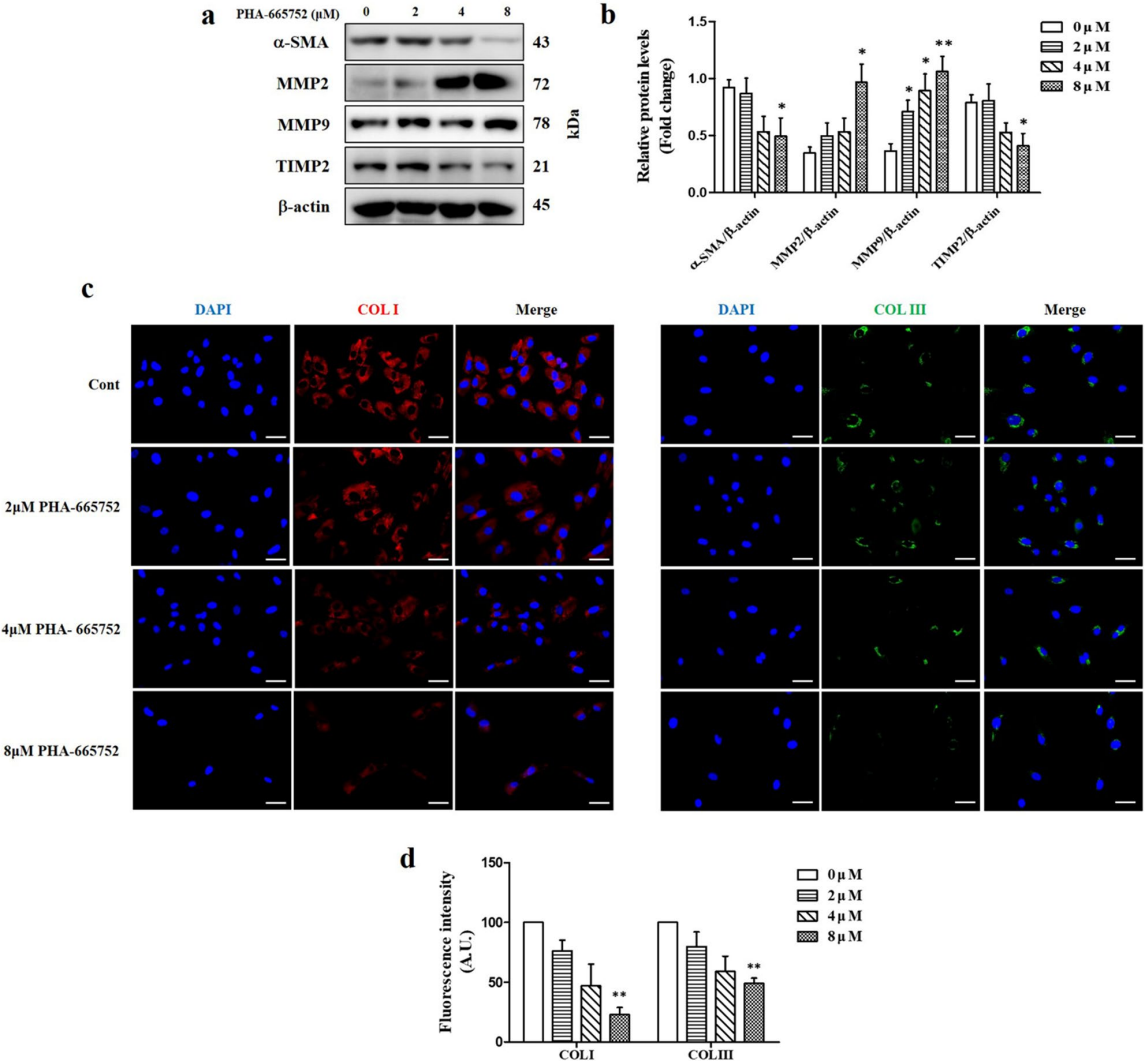
Effects of PHA-665752 on collagen accumulation and ECM synthesis. (**a**) Western blot analysis of the expression of α-SMA, MMP2, MMP9, and TIMP2 in keloid fibroblasts treated with PHA-665752. Full-length blot images are presented in Supplementary Fig. 4a. (**b**) The relative protein levels of α-SMA, TIMP2, MMP2, and MMP9 (*represents *p* < 0.05, **represents *p* < 0.005). (**c**) Immunofluorescence for COL I and COL III. Nuclei were stained with DAPI (blue) (original magnification: 200 × , scale bar = 50 μm). (**d**) Fluorescence intensity of COL I and COL III (**represents* p* < 0.005). Cont, control.

The cells treated with 0, 2, 4, and 8 μM PHA-665752 for 24 h (Fig. 4a). Alpha smooth muscle actin (α -SMA) expression levels were 2.5 times lower in cells treated with 8 μM PHA-665752 (0.36 ± 0.37) than those in the controls (0.92 ± 0.17). Tissue inhibitor of metalloproteinase 2 (TIMP2) expression levels were 1.9 times lower in cells treated with 8 μM PHA-665752 (0.41 ± 0.24) than those in the controls (0.79 ± 0.14). In contrast, the expression level of metalloproteinase (MMP) 2 was significantly higher after treatment with 8 μM PHA-665752 (0.97 ± 0.35) than that in the controls (0.35 ± 0.12). MMP9 expression levels were also elevated after treatment with 2, 4, and 8 μM of PHA-665752 (0.71 ± 0.2, 0.89 ± 0.29, and 1.06 ± 0.3, respectively) compared with those in the control group (0.34 ± 0.14) (Fig. 4b). Immunofluorescence revealed lower collagen type I and type III expression in cells treated with 8 μM PHA665752 (22.78% ± 10.5%, and 49.03% ± 7.87% lower, respectively) than that in controls (Fig. 4c,d).

### Effects of PHA-665752 in a human keloid fibroblast-derived SCID mouse model of nodule formation

**Figure 5:**
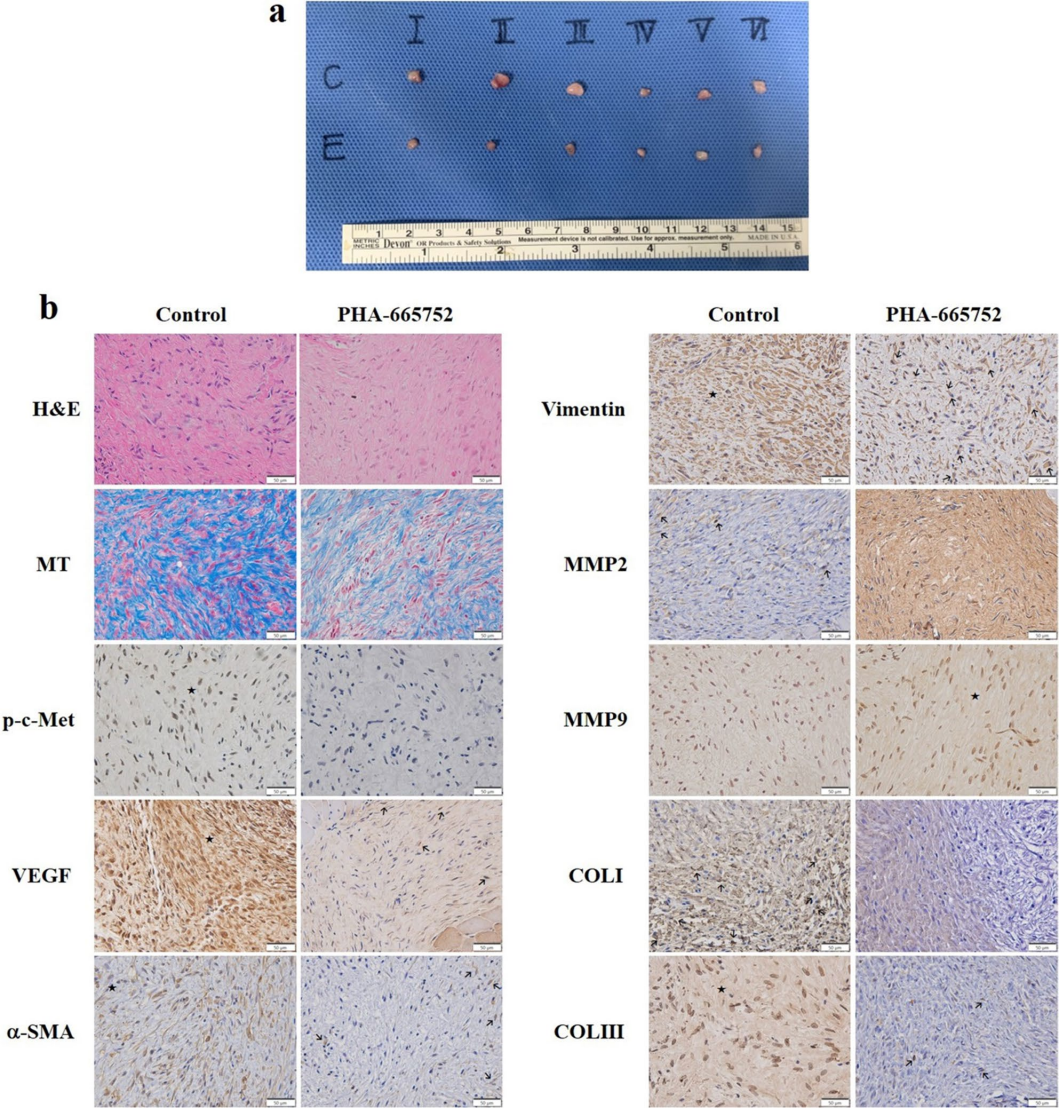
Histological and immunohistochemical images of the nodules formed by human keloid fibroblasts in SCID mice 7 days after PHA-665752 treatment. (**a**) Nodules were harvested from mice treated with DMSO or PHA-665752. (**b**) Representative H&E staining, MT staining, and immunohistochemistry for the expression of p-c-Met, VEGF, α-SMA, vimentin, MMP2, MMP9, COL I, and COL III in the nodules. The arrows (individual cells) and star (most cells) represent positive (brown) expression. (Original magnification: 400 × , scale bar = 50 μm).

On day 7, the SCID mouse group treated with PHA-665752 had an average nodule volume of 12.07 ± 5.18 mm^3^, whereas the group treated with 2% DMSO had an average nodule volume of 30.16 ± 8.51 mm^3^. PHA-665752 markedly suppressed nodule volume compared with 2% DMSO (Fig. 5a and Supplemental Fig. 1a). The average nodule weight was 2.3 times lower in the PHA-665752–injected group (0.01 ± 0.00 g) than that in the 2% DMSO-injected group (0.03 ± 0.02 g) (Supplemental Fig. 1b). Seven days after PHA-665752 injection, the PHA-665752 group had significantly fewer inflammatory cells per 0.1 mm^2^ (15.75 ± 5.32) than those in the 2% DMSO group (30 ± 1.83 cells) (Fig. 5b and Supplemental Fig. 1c). MT staining revealed significantly lower collagen content in the PHA-665752–treated group (37.13% ± 6.12%) than that in the 2% DMSO-treated group (69.01% ± 10.6%) (Fig. 5b and Supplemental Fig. 1d). We observed significantly more cells expressing p-c-Met, VEGF, α-SMA, vimentin, collagen type I, and collagen type III in the 2% DMSO group (7.24% ± 3.29%, 45.7% ± 9.88%, 27.87% ± 10.21%, 59.42% ± 8.51%, 47.12% ± 10.59%, and 27.03% ± 10.87%, respectively) than those in the PHA-665752 group (1.14% ± 0.46%, 41.43% ± 5.4%, 17.2% ± 3.8%, 51.33% ± 7.45%, 21.99% ± 7.09%, and 8.58% ± 5.21%, respectively). MMP2 and MMP9 were expressed in lower percentages of cells in the 2% DMSO-treated group (41.41% ± 6.05% and 43.61% ± 11.83%, respectively) than those in the PHA-665752–treated group (57.26% ± 12.48% and 63.32% ± 12.54%, respectively) (Fig. 5b and Supplemental Fig. 1e).

## Discussion

We observed increased collagen synthesis and overexpression of c-Met in keloid specimens, consistent with the results of previous studies. Upon treatment with PHA-665752, we observed lower keloid fibroblast viability than that in untreated samples. PHA-665752 functions by inhibiting HGF-stimulated c-Met autophosphorylation and the constitutive phosphorylation of downstream mediators of the HGF/c-Met pathway in multiple tumor cell lines^14^. These results suggest that PHA-665752 is a potent inhibitor of c-Met activity in keloid scars and interferes with the downstream HGF/c-Met pathway^14^. scratch test demonstrated that PHA-665752 inhibited keloid fibroblast migration. Since fibroblast migration is known to be mediated by TGF-β activation, our findings are consistent with a previous study that suggested PHA-665752 has an inhibitory effect on TGF-β signaling^17^.

After we determined the inhibitory effects of PHA-665752 on fibroblast survival, proliferation, and migration, we examined its effects on the expression of proteins that are involved in the formation of keloid scars. TIMP2 is an endogenous inhibitor of MMPs. High levels of TIMP2 lead to accumulation of the extracellular matrix (ECM), whereas low levels of TIMP lead to matrix proteolysis We found higher levels of MMPs and a lower TIMP2-to-β-actin ratio in cells treated with PHA-665752 than those in control cells. Although MMPs can be elevated during the wound-healing process^18^, concurrent MMP elevation and TIMP suppression suggest that PHA-665752 enhances MMP expression, resulting in less ECM accumulation. Consistent with this interpretation, the c-Met inhibitor also suppressed collagen synthesis.

Given that keloids occur exclusively in humans, animal models have been difficult to develop and are rarely used^19^. We used the keloid animal model that was introduced by Fujiwara et al., who implanted keloid fibroblasts into the dorsal skin of SCID mice^20^. We found that nodule size in both the PHA-665752 injection and DMSO groups decreased over time; nodule volume decreased more quickly in the inhibitor group. Although it is unclear why the keloid nodules decreased in size even in the untreated group, it might have been caused by a natural regression, as keloids occur exclusively in humans. This phenomenon was also observed in the study by Fujiwara et al., but it is not typically observed in human keloid tissues. Keloid regression contributes to the difficulty of producing animal keloid models and implies that the treatment response may differ from a potential clinical response. However, the weights of the harvested nodules differed between the treatment and control groups, which suggested that PHA-665752 antagonized keloid tissue formation or maintenance.

Our study has several limitations. First, the in vivo model does not accurately represent keloid pathogenesis. Second, although the site of origin may affect keloid tissue responses to PHA-665752, we did not analyze our results based on tissue origin. Finally, more keloid tissue samples would have contributed to a more generalizable, reliable result.

## Conclusion

PHA-665752, a selective small-molecule inhibitor of c-Met, may be an alternative treatment modality that effectively reduces the proliferation of keloids. In the future, additional experimental research and clinical trials will be needed to test the safety and effectiveness of c-Met inhibitors for the treatment of keloid scars. A combination therapy comprising PHA-665752 with other effective keloid treatment modalities may improve patient outcomes.

## Methods

### Ethical approval

The study has been reviewed and approved by Boramae Hospital Institutional Review Board in compliance with Helsinki Declaration and Integrated Addendum to ICH: Guideline for Good Clinical Practice (ICH-GCP) (approval no. 26-2017-20). Samples of human keloid tissue were obtained from patients who provided informed consent prior to conducting this study according to the guidelines of the Ethics Committee.

### Human keloid-derived fibroblast cell cultures

Keloid tissues were obtained from six patients who underwent excision surgery at Seoul National University Boramae Hospital. The keloid tissues were washed three times with Dulbecco’s phosphate-buffered saline with 1% antibiotic–antimycotic solution (A/A; Welgene, Gyeongsangbuk-do, Republic of Korea) and cut into 3-mm-thick pieces. Subsequently, the tissues were digested with 5 mg/ml dispase (Roche, Basel, Switzerland) for 4 h at 37 °C. The dermis and epidermis were separated, cut into 1-mm-thick pieces, and digested to a single-cell suspension with 3 mg/ml collagenase type I (Thermo Fisher, Waltham, MA, USA). The cells were growth in Dulbecco’s modified Eagle’s medium (DMEM; Biowest, Nuaillé, France) containing 10% fetal bovine serum (FBS; Biowest, France) and 1% A/A in a humidified incubator with 5% CO_2_ at 37 °C. Cells from passage 3 to 5 were used for experiments.

### Cell viability analysis

Cell viability was measured using Cell Counting Kit-8 (CCK-8; Dojindo Laboratories, Kumamoto, Japan). Keloid fibroblasts (1.0 × 10^4^ cells/well) were cultured in triplicate in DMEM containing 10% FBS and 1% A/A in 96-well plates. The cells were treated with PHA-665752 (0.1, 0.2, 0.4, 0.8, 1, 2, 4, 6, 8, and 10 μM; Selleckchem, Houston, TX, USA) for 24 h. Then, a mixture of CCK-8 solution (10 μL) and DMEM (90 μL) was added to each well, and the plates were incubated for 1 h in an incubator at 37 °C in 5% CO_2_. The absorbance of each well was measured at a wavelength of 450 nm with a SpectraMax ABS (Molecular Devices, San Jose, CA, USA).

### In vitro western blot analysis

Human keloid fibroblasts were lysed with radioimmunoprecipitation assay buffer (Thermo Fisher, Waltham, MA, USA), and the protein concentration was measured using a BCA Protein Assay Kit (Thermo Fisher, Waltham, MA, USA). Equal amounts of protein were loaded on 8% and 12% sodium dodecyl sulfate–polyacrylamide gels and, after electrophoresis, transferred to polyvinylidene fluoride (PVDF) membranes (Millipore, Boston, MA, USA). After the PVDF membranes were blocked with 5% bovine serum albumin (BSA) in Tris-buffered saline containing TWEEN Registered 20, they were incubated overnight at 4 °C with primary antibodies diluted in blocking buffer: phospho-c-Met (Tyr1234/1235, 1:1,000), c-Met (N-terminal, 1:1,000), phospho-mTOR (p-mTOR; Ser2448, 1:1,000), mTOR (7C10, 1:1,000), phospho-p44/42 MAPK (p-p44/42 MAPK; Thr202/Tyr204, 1:1,000), p44/42 MAPK (Erk1/2, 1:1,000), ɑ-SMA (1:1,000), MMP2 (1:1,000), MMP9 (1:1,000), TIMP2 (3A4, 1:1,000) and β-actin (1:5,000). Then, the membranes were incubated for 1 h at room temperature with horseradish peroxidase (HRP)-conjugated secondary antibodies: goat anti-rabbit IgG HRP (1:5,000) and goat anti-mouse IgG HRP (1:5,000). Protein bands were detected by chemiluminescence using a SuperSignal ECL kit (Thermo Fisher, Waltham, MA, USA) and visualized using an ImageQuant LAS 4000 (GE Healthcare Life Science, Marlborough, MA, USA).

### Immunofluorescence

All samples were fixed in 4% paraformaldehyde overnight, permeabilized with 100% methanol for 2 min, and blocked in 5% BSA for 1 h at room temperature. Samples were incubated with primary antibodies against collagen I (Col I, 1:1,000) and collagen III (Col III, 1:1,000) diluted in a blocking buffer overnight at 4 °C. The samples were then stained with secondary antibodies (Alexa Fluor Registered 594 goat anti-mouse IgG [1:200] and Alexa Fluor Registered 488 goat anti-rabbit IgG [1:200]) for 1 h, and nuclei were stained with 1 μg/ml NucBlue Live Cell Stain ReadyProbes reagent (Thermo Fisher, Waltham, MA, USA) for 20 min. The samples were scanned using fluorescence inverted microscopy (Leica Microsystems, Wetzlar, Germany). Fluorescence intensity was measured using ImageJ 1.36b imaging software (National Institutes of Health, Bethesda, MD, USA).

### Migration assay

Human keloid fibroblasts (2.0 × 10^4^) were seeded in a 24-well plate. After the cells attached to the plate, we created a straight-line scratch in each well using a 1-ml pipette tip. The cells were then treated with PHA-665752 (0, 2, 4, and 8 μM) in DMEM containing 2% FBS and 1% A/A for 24 h. Images of the wound gap area at the initial time (0 h) and 24 h after the scratching were captured under an inverted fluorescence microscope (Leica Microsystems, Wetzlar, Germany). The area of the gap created by the scratch was calculated using ImageJ software and then compared to the size of the initial gap.

### Experimental animals

The animal study was approved by the Institutional Animal Care and Use Committee of the Seoul National University Boramae Hospital (approval no. 2019–0031). Male NOD.CB17-Prkdc scid/J (SCID) mice (5 weeks old, body weight: 22.58 ± 0.91 g) were purchased from Charles River Japan (Yokohama, Japan). All animal experimental procedures were performed in accordance with ARRIVE guidelines^21^ and the Seoul National University Hospital Institutional Animal Care and Use Committee guidelines.

### Nodule formation by cultured human keloid fibroblasts in SCID mice

All experiments were performed under aseptic conditions. Under anesthesia using isoflurane, we removed the dorsal surface hair from 6 SCID mice and marked the middle line, and then subcutaneously injected 150 μL human keloid fibroblasts (5.0 × 10^8^ cells) through a 1-ml syringe (Becton, Dickinson and Company, Franklin Lakes, NJ, USA) with a 26-gauge needle. These cells were obtained from six patients who underwent excision surgery at in our hospital. The cells were cultured 1 cm away from the left and right sides of the most prominent region of the mouse backs. After nodules formed on the backs of the mice, we injected the left nodules with 2% dimethyl sulfoxide (DMSO; control) and the right nodules with PHA-665752 (16.5 μg/100 μL). The nodules were measured every 2 to 3 days with calipers, and nodule volume was calculated using the equation: Nodule volume = 1/2 × A × B^2^, where A = length in millimeters and B = width in millimeters^15^. On day 14 after fibroblast injection, to harvest the nodules, the surrounding skin was carefully separated used forceps and a blade, and only the nodules were collected and weighed.

### In vivo histological analysis and immunohistochemistry

The nodules were harvested 14 days after the initial cell injection, and then fixed in 4% paraformaldehyde for 24 h at 4 °C, washed with water for at least 4 h, and finally embedded in paraffin. The paraffin sections (4 µm thick) were stained with hematoxylin and eosin (H&E, ab245880, Abcam, Cambridge, UK) and MT (TRM-2, ScyTek, Logan, Utah, USA) stain, according to the manufacturers’ guidelines. The cellularity in the H&E-stained sections (400 × magnification) and collagen density in the MT-stained sections (400 × magnification) were confirmed for each section using an Olympus BX53 microscope (Olympus Corporation, Shinjuku, Japan); we acquired screenshots of three microscopic fields (right, center, and left). The number of inflammatory cells per unit area (0.1 mm^2^) was calculated using the Olympus cellSens standard imaging software. The optical density of collagen was measured using ImageJ 1.36b.

Immunohistochemical analysis was performed using the avidin–biotin complex method. Briefly, sections were deparaffinized and incubated with trypsin enzymatic antigen retrieval solution (ab970; Abcam, Cambridge, UK) for 30 min at 37 °C. Sections were incubated overnight at 4 °C with primary antibodies: p-c-Met (1:20), VEGF (1:50), α-SMA (1:300), vimentin (1:400), MMP2 (1:200), MMP9 (1:50), Col I (1:500), and Col III (1:200). After washing three times with Tris-buffered saline containing 0.025% Triton X-100, the sections were incubated with secondary antibodies for 30 min at room temperature. Images (right, center, and left) were captured with an Olympus BX53 microscope. The optical density of each image was quantified using ImageJ 1.36b imaging software.

### Antibodies

The following primary antibodies were used for western blotting, immunofluorescence, and immunohistochemical analysis: p-c-Met (#3077; Cell Signaling Technology, Danvers, MA, USA), c-Met (ab51067; Abcam, Cambridge, UK), p-mTOR (#2971; Cell Signaling Technology), mTOR (#2983; Cell Signaling Technology), p-p44/42 MAPK (#9101; Cell Signaling Technology), p44/42 MAPK (#9102; Cell Signaling Technology), α-SMA (14,395-1-AP; Proteintech, Rosemont, IL, USA), MMP2 (10,373-2-AP; Proteintech), MMP9 (10,375-2-AP; Proteintech), TIMP2 (sc-21735; Santa Cruz Biotechnology, Dallas, Texas, USA), COL I (immunofluorescence: ab6308; Abcam; immunohistochemistry: LS-C343921; LSBio, Seattle, WA, USA), COL III (immunofluorescence: ab7778; Abcam; immunohistochemistry: LS-C413514; LSBio), β-actin (sc-47778; Santa Cruz Biotechnology), VEGF (sc-57496; Santa Cruz Biotechnology), and vimentin (ab92547; Abcam). The following secondary antibodies were used: anti-rabbit IgG HRP (western blotting: #7074; Cell Signaling Technology: immunofluorescence: R37117; Thermo Fisher, Waltham, MA, USA; immunohistochemistry: PK-4001; Vector Laboratories, Burlingame, CA, USA) and goat anti-mouse IgG HRP (western blotting: ADI-SAB-100; Enzo Life Science, Farmingdale, NY, USA; immunofluorescence: A11005; Thermo Fisher; immunohistochemistry: PK-4002; Vector Laboratories).

### Statistical analysis

In vitro experimental results are reported as the mean value ± standard deviation of at least 3 independent experiments. For in vivo experiments, all values are reported as the mean ± standard error of the mean. Statistical tests were performed with GraphPad Prism 5.0 software. Statistical significance was determined using the Mann–Whitney nonparametric U test, and multiple comparisons were tested with two-way analysis of variance. A *p* value less than 0.05 was considered as statistically significant.

## Supplementary Information

Supplementary Information

## Abbreviations

A/A: Antibiotic–antimycotic solution
α-SMA: Alpha smooth muscle actin
BSA: Bovine serum albumin
CCK-8: Cell counting kit-8
c-Met: C-mesenchymal-epithelial transition factor
DMEM: Dulbecco’s modified Eagle’s medium
FBS: Fetal bovine serum
ECM: Extracellular matrix
ERK: Extracellular signal-regulated kinases
HGF: Hepatocyte growth factor
PVDF: Polyvinylidene fluoride
HRP: Horseradish peroxidase
MAPK: Mitogen-activated protein kinase
MMP: Matrix metalloproteinase
MT: Masson’s trichrome
mTOR: Mammalian target of rapamycin
PI3K: Phosphatidylinositol 3-kinase
SCID: Severe combined immunodeficient
TGF-β: Transforming growth factor-β
TIMP: Tissue inhibitor of metalloproteinase
VEGF: Vascular endothelial growth factor
