# Supplementary material for: A selective small-molecule inhibitor of c-Met suppresses keloid fibroblast growth in vitro and in a mouse model
Source: Sci Rep. 2021 Mar 9;11:5468. doi: 10.1038/s41598-021-84982-4 (PMC7943593; doi:10.1038/s41598-021-84982-4)
Supplement: Supplementary file 1 — Supplementary Information [file 41598_2021_84982_MOESM1_ESM.docx]

**Title:** A selective small-molecule inhibitor of c-Met suppresses keloid fibroblast growth *in vitro* and in a mouse model

Min-Ha Choi, MS^1*^, Jinhyun Kim, MD^1*^, Jeong Hyun Ha, MD^1^, Ji-Ung Park, MD, PhD^1^

^1^Department of Plastic and Reconstructive Surgery, Seoul National University Boramae Hospital, Seoul National University College of Medicine, Seoul, Republic of Korea

* Min-Ha Choi and Jinhyun Kim contributed equally to this study as first authors.

**Corresponding author:**

Ji-Ung Park, MD, PhD

Department of Plastic and Reconstructive Surgery

Seoul National University Boramae Hospital

Seoul National University College of Medicine

5 Gil 20, Boramae-Road, Dongjak-Gu, 07061, Seoul, Republic of Korea

E-mail: [alfbskan@gmail.com](mailto:alfbskan@gmail.com)

Tel: +82-2-870-2332

Fax: +82-2-831-2826

ORCID ID:0000-0002-6403-4918

**Key words:** Keloid, c-Met receptor tyrosine kinase, PHA-665752, hepatocyte growth factor pathway

**Supplementary contents**


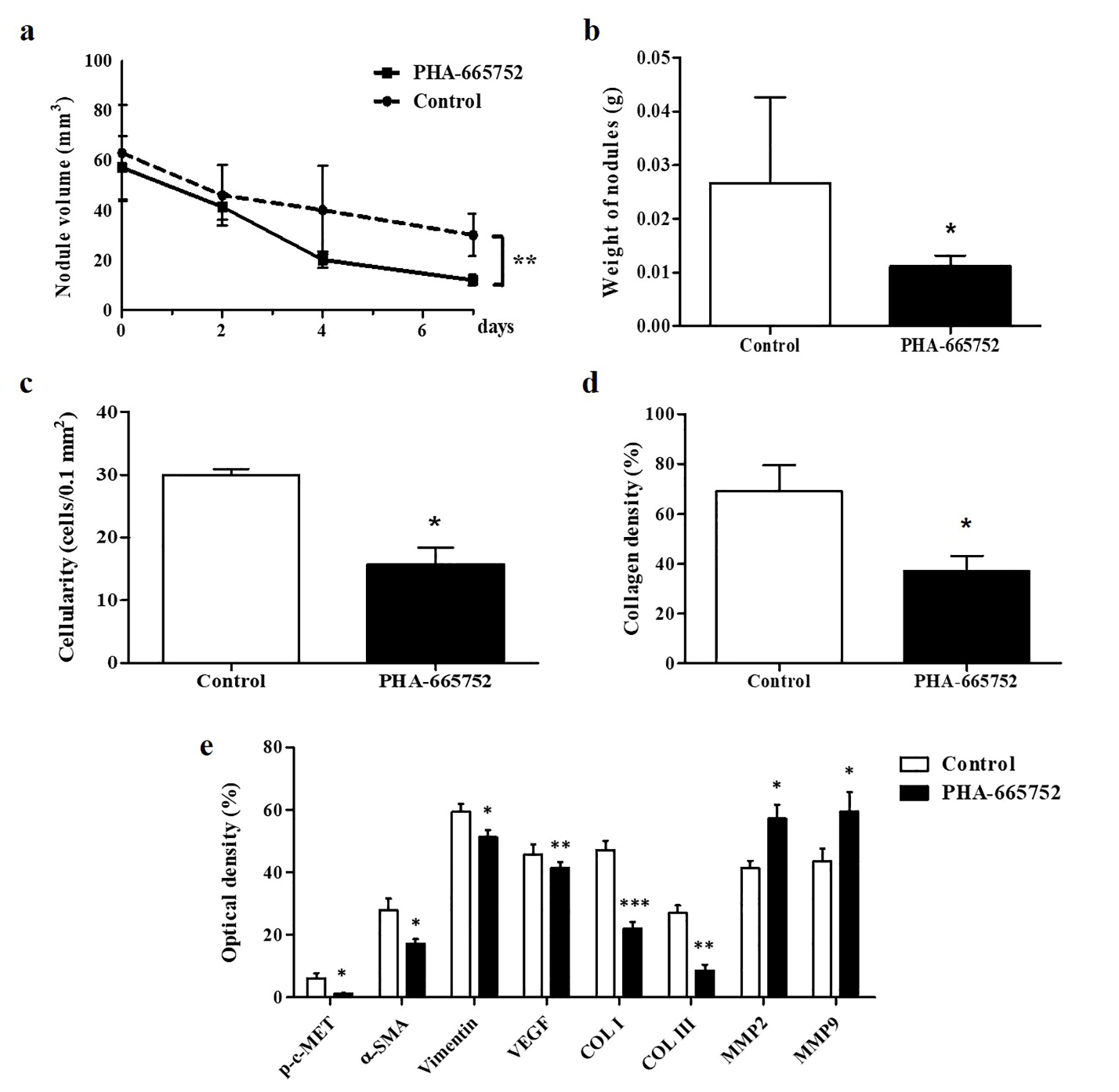


**Supplemental figure 1.** **Effect of PHA-665752 on nodule formation.** **a**, Human keloid-derived fibroblasts were subcutaneously injected into severe combined immunodeficient (SCID) mice. Three days after verification of nodule formation, 2% dimethyl sulfoxide (DMSO) or 16.5 μg/100 μl PHA-665752 was injected once into each nodule. Nodule volume was monitored for 7 days (** represents *p* < 0.005). **b**, Nodule weight (* represents *p* < 0.05). **c**, Quantitative analysis of cellularity (* represents *p* < 0.05). **d**, Collagen density (* represents *p* < 0.05). **e**, Quantitative analysis of phosphorylated c-mesenchymal-epithelial transition factor (p-c-Met), vascular endothelial growth factor (VEGF), alpha smooth muscle actin (α-SMA), vimentin, matrix metalloproteinase (MMP) 2, MMP9, collagen type I (COL I), and collagen type III (COL III) expression in nodules harvested from SCID mice (* represents *p* < 0.05, ** represents *p* < 0.005, and *** represents *p* < 0.0001).


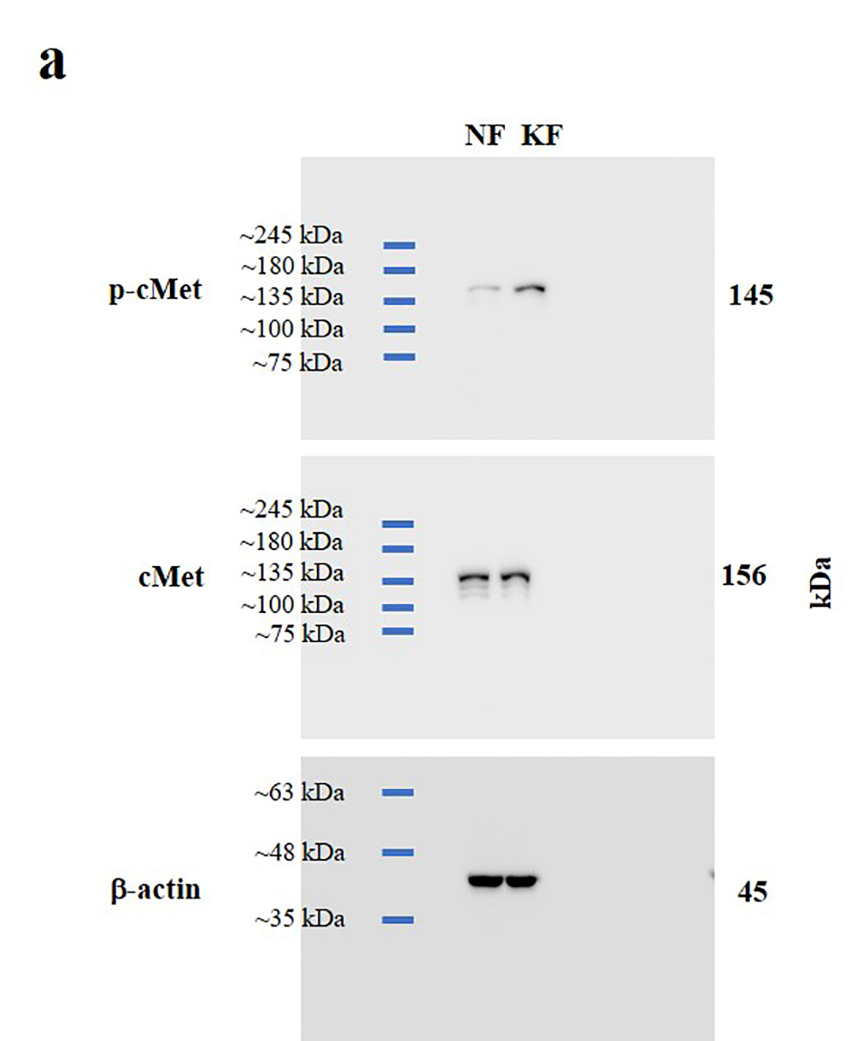


**Supplemental figure 2.** **Full-length blot images. a,** Figure 1d


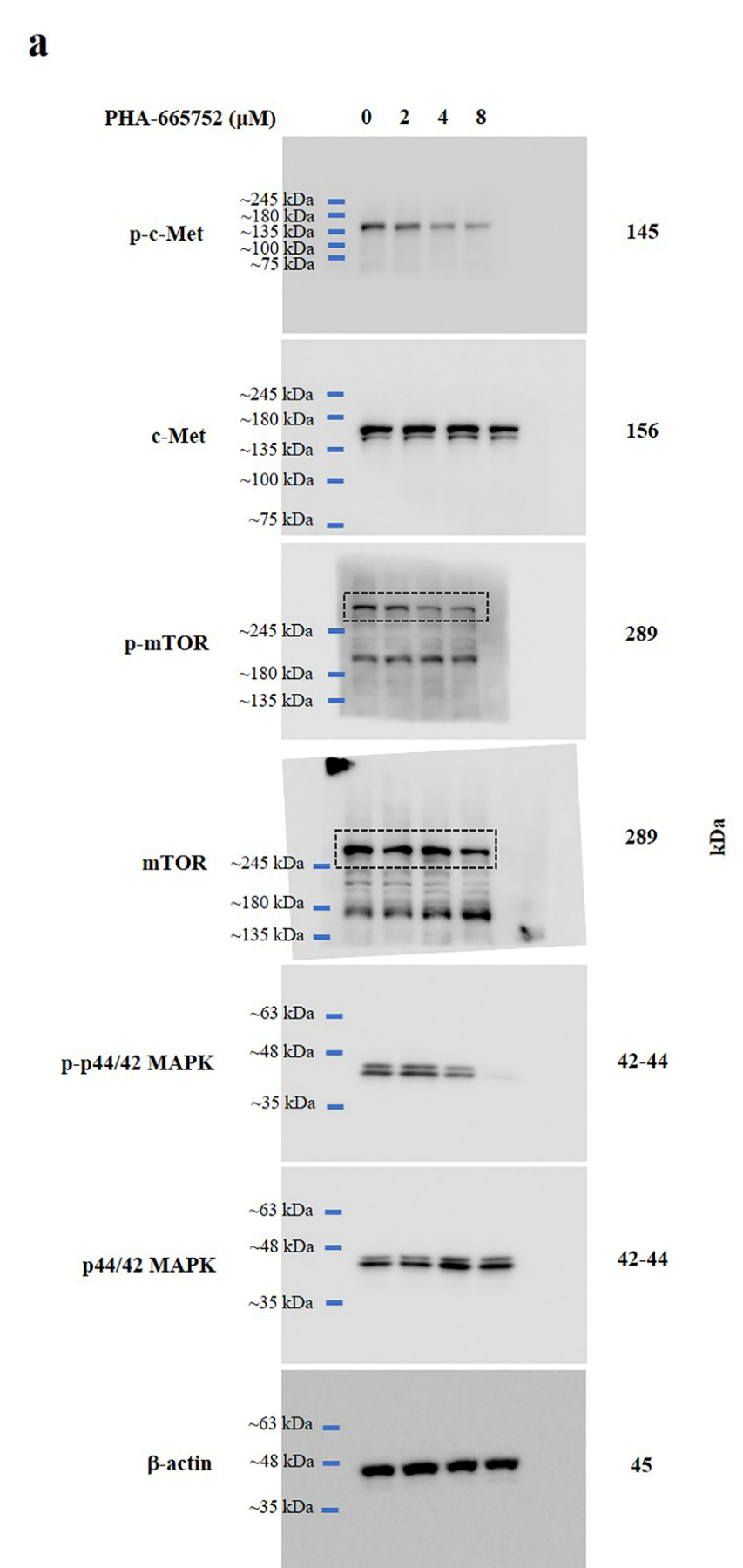


**Supplemental figure 3.** **Full-length blot images. a,** Figure 2a (Dashed boxes represents the images included in the figures)


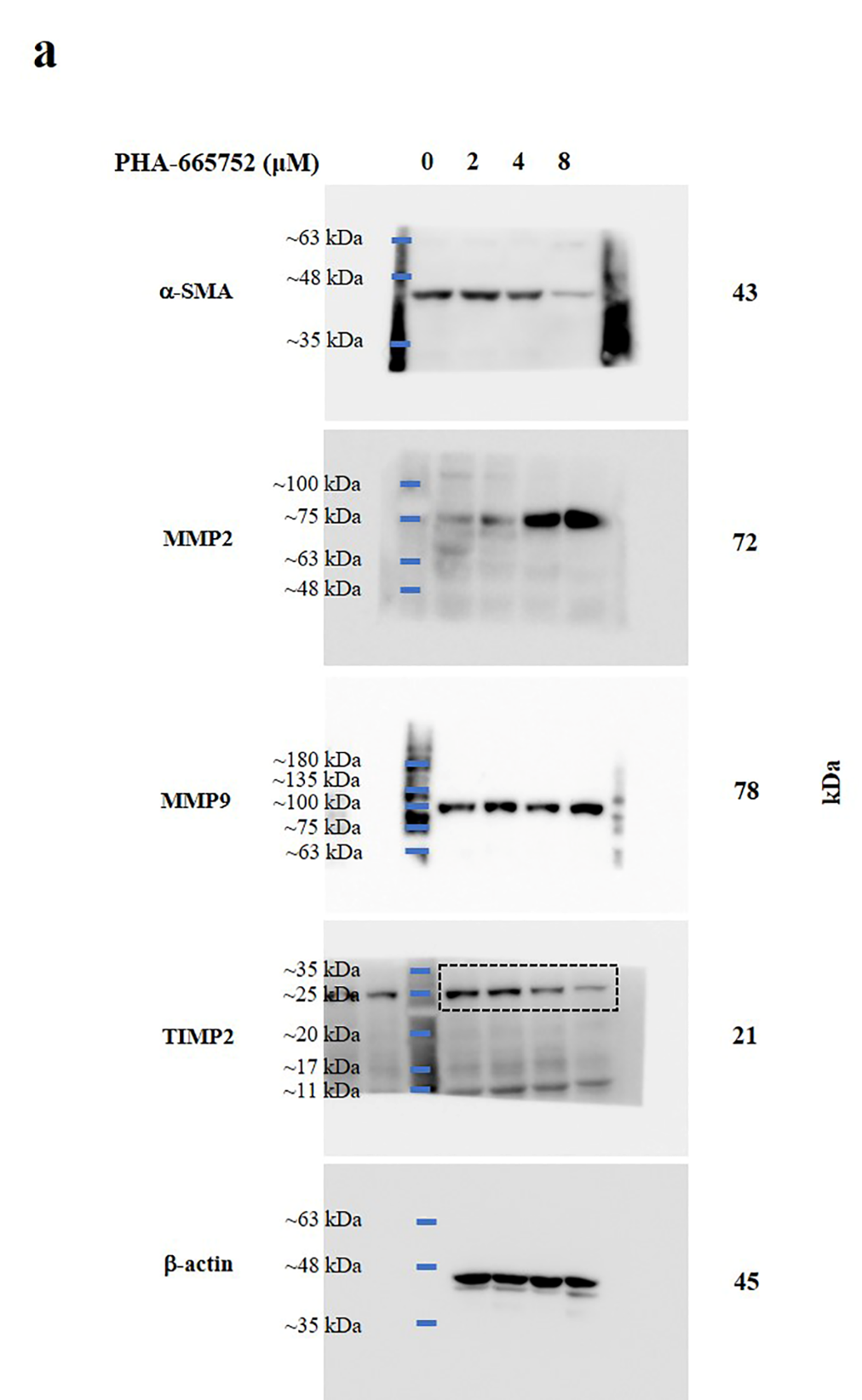


**Supplemental figure 4.** **Full-length blot images. a,** Figure 4a (Dashed boxes represents the images included in the figures)
